# Association of atopic dermatitis and headache disorder: a systematic review and meta-analyses

**DOI:** 10.3389/fneur.2024.1383832

**Published:** 2024-03-21

**Authors:** Wei Yang, Hong Dai, Xiao-feng Xu, Hai-yin Jiang, Ji-yuan Ding

**Affiliations:** ^1^Department of Oncology, Hangzhou Red Cross Hospital, Hangzhou, Zhejiang, China; ^2^State Key Laboratory for Diagnosis and Treatment of Infectious Diseases, Collaborative Innovation Center for Diagnosis and Treatment of Infectious Diseases, The First Affiliated Hospital, College of Medicine, Zhejiang University, Hangzhou, China

**Keywords:** allergic, atopic, eczema, cephalgia, cephalalgia

## Abstract

**Background:**

Growing evidence suggests that headache disorders and atopic dermatitis share similar pathological mechanisms and risk factors. The aim of this study was to assess the risk for headache disorders in patients with atopic dermatitis.

**Methods:**

We systematically searched the PubMed and Embase databases from inception to December 1, 2023, for observational studies that examined risk of migraine in subjects with atopic dermatitis. Risk estimates from individual studies were pooled using random-effects models.

**Results:**

Ten studies with 12,717,747 subjects were included in the meta-analysis. Our results showed that patients with atopic dermatitis were associated with a higher risk of headache disorder (OR, 1.46, 95% CI = 1.36–1.56; *P* < 0.001; I^2^ = 98%) or migraine (OR, 1.32, 95% CI = 1.18–1.47; *P* < 0.001; I^2^ = 98.9%). Most of the results of the subgroup analyses were consistent with the overall results.

**Conclusion:**

The findings of this meta-analysis suggest that atopic dermatitis is a potential risk indicator for headache disorder or migraine. Further studies are still needed to verify our findings due to the substantial heterogeneity in our analyses.

## Introduction

Headaches, including migraine, tension-type headaches, and cluster headaches, are among the most prevalent neurological disorders associated with pain sensation, and significantly diminish patients' quality of life (1). Approximately 52% of the global population experience a headache disorder annually (2). Factors such as family history, smoking, alcohol consumption, male predominance, and head trauma are known to predict the occurrence of headaches (3). Early identification and treatment of these risk factors are crucial for high-risk patients susceptible to early-onset headaches. Additionally, several chronic inflammatory conditions such as systemic lupus erythematosus (4), inflammatory bowel disease (5), and chronic periodontitis (6) are recognized as significant risk factors for headaches.

In recent years, atopic diseases have emerged as a growing global public health concern, affecting ~20–40% of the population worldwide, with incidence rising sharply in developing countries (7). Atopic dermatitis (AD) is the most common chronic condition in early childhood and often marks the start of the atopic march: the progression from AD in infancy to allergic rhinitis and asthma in later childhood (8, 9). As an inflammatory response mediated by diverse immunological pathways (10), AD warrants closer examination in patients with headaches. While several prior systematic reviews have established a positive link between asthma and migraines (11–13), studies focusing specifically on AD have yielded inconclusive results. Subsequent to these reviews, new research has begun to investigate the potential relationship between AD and headaches (14–23). The connection between AD and headache disorders is not yet fully understood, but the increasing number of reports on AD in patients with headaches has raised concerns among healthcare providers. This study aimed to review systematically the available evidence on the association between AD and the onset of headaches, and to estimate the strength of this association through a meta-analysis.

## Methods

### Search strategy

This systematic review followed the Preferred Reporting Items for Systematic Reviews and Meta-Analyses (PRISMA) guidelines. A thorough search of the PubMed and Embase databases was conducted, covering all publications from their inception up to December 1, 2023. The search terms used were “(dermatitis OR atopic dermatitis OR eczema) AND (migraine OR hemicrania OR headache OR cephalgia OR cephalalgia)” with no restrictions on language. Additionally, the reference lists of the selected articles and related reviews were scrutinized to identify pertinent articles not captured in the electronic search.

### Inclusion criteria

The criteria for inclusion in this review were as follows: (1) the study design was either cross-sectional, case-control, or cohort; (2) the study investigated the association between AD and the risk of headaches; (3) the study provided risk estimates with confidence intervals (CIs) or sufficient data to calculate these estimates; (4) the data were published in a complete text format; and (5) the study had a sample size > 1,000. Exclusions were made for case reports, animal studies, editorials, correspondences, and reviews.

### Data extraction and quality assessment

Data extraction was independently performed by two double-blinded authors. The extracted data included the first author's name, publication year, study design, location, subjects, methods of AD and headache ascertainment, adjustments for confounders, results, and quality. The quality of the included studies was evaluated using the Newcastle-Ottawa Scale (24), as recommended by the Cochrane Collaboration. This scale comprises eight items and assigns scores ranging from 0 (indicating a high risk of bias) to 9 (indicating a low risk of bias). Studies scoring ≥ 7 were deemed high quality. One author conducted the initial details and assessment of the included studies, which was then reviewed by another author for accuracy. Any discrepancies were resolved through discussion.

### Statistical analysis

Statistical analysis was conducted using STATA 10.0 software (StataCorp LP, College Station, TX, United States). To account for clinical heterogeneity among studies, due to variations in study designs and measurement criteria, the random-effects model was applied in this meta-analysis (25). Heterogeneity among the included studies was quantified using the I^2^ statistic. Heterogeneity levels were interpreted as follows: I^2^ < 25% indicated an absence of heterogeneity; 25% ≤ I^2^ < 50% suggested low heterogeneity; 50% ≤ I^2^ < 75% represented moderate heterogeneity; and I^2^ ≥ 75% indicated substantial heterogeneity (26). We either extracted the most fully adjusted effect estimates (ORs, RRs, or HRs) or calculated the unadjusted OR using raw data. The association between AD and headache risk was estimated using odds ratios (ORs) and their corresponding 95% CIs, derived from comparisons between cases and controls (27). To assess publication bias, Egger’s regression test was used for quantitative evaluation, and funnel plots of the logarithm of OR versus the standard error were examined for qualitative assessment (28, 29). *P* < 0.05 was considered statistically significant (Table 1).

**Table 1:** Characteristics of the included studies.

Author, year	Study population	Study design/period	Target group	Atopic disease measurement	Headache assessment	Number of subjects	Adjustment	Nos. quality
Silverberg (14)	National Health Interview Survey in USA	Cross-sectional/1997-2013	Children and adolescents	Self-reported diagnosis by questionnaire	Self-reported diagnosis by questionnaire	401,002	Age, sex, race/ethnicity, household income, highest level of education in the family, insurance coverage, number of persons in the household, birthplace in the United States, ever history of asthma, hay fever, and food allergy	6
Shreberk-Hassidim et al. (15)	Israeli civilians undergo a routine medical evaluation and fitness-for-service	Cross-sectional/1998-2003	Adolescents (16-21 years)	Medical record review	Medical record review	1,187,757	Age, region of birth, predicted socioeconomic status (SES), and comorbidities	6
Wei et al. (16)	National Health Insurance in China	Case-control	Children (7-18 years)	ICD-9	ICD-9	16,130/64,520	Comorbidity	7
Smirnova et al. (17)	Life and Health (Liv och Hälsa) in Sweden	Cross-sectional/2007	Adults (>20 years)	Self-reported diagnosis by questionnaire	Self-reported diagnosis by questionnaire	34313	Sex, age group, smoking, and education level	6
Manjunath and Silverberg (18)	The Fragile Families and Child Wellbeing Study in USA	Case-control/1998-2015	Adolescents (15 years)	Parent questionnaire	Parent questionnaire	4898	Sex and race/ethnicity	5
Roh et al. (19)	IBM MarketScan Commercial Claims and Encounters database in USA	Case-control/1998-2015	Adults (>18 years)	ICD-10-CM	ICD-10-CM	39,779/353,743	Age, sex, and number of outpatient encounters	7
Fan et al. (20)	National Institutes of Health database in USA	Cross-sectional	Adults (≥18 years)	ICD-9-CM or ICD-10-CM	ICD-9-CM or ICD-10-CM	12,008/202,118	Age, sex, race/ethnicity, smoking status, body mass index, hypertension, hyperlipidemia, cardiovascular disease, Type 2 diabetes mellitus, sleep apnea, and hypothyroidism	8
Fuxench et al. (21)	The Health Improvement Network in UK	Cohort/NA	Child and adult	Diagnostic codes and 2 AD-treatment codes	Diagnostic codes	1,034,514/2,219,898	Age, sex, Townsend index, history of allergic rhinitis, asthma, depression, anxiety, and hormonal medications	8
Han et al. (22)	Korean National Health Insurance Service database	Cohort/2009	Adults (>20 years)	ICD-10-CM	ICD-10-CM	3,607,599	Age, sex, smoking status, alcohol consumption, physical activity, hypertension, diabetes mellitus, dyslipidemia, myocardial infarction, stroke, anxiety disorder, bipolar disorder, and depression	8
Lee et al. (23)	Korean National Health Insurance Service (K-NHIS) claims database	Cohort/2009	Adults (≥18 years)	Least one diagnosis with prescription of the topical agent or three or more occasions of diagnosis with dedicated ICD-10 codes	ICD-10	48,983/3,490,485	Age, sex, smoking status, alcohol consumption, physical activity, hypertension, diabetes mellitus, dyslipidemia, myocardial infarction, stroke, anxiety disorder, bipolar disorder, and depression	8

## Results

### Search results

**Figure 1:**
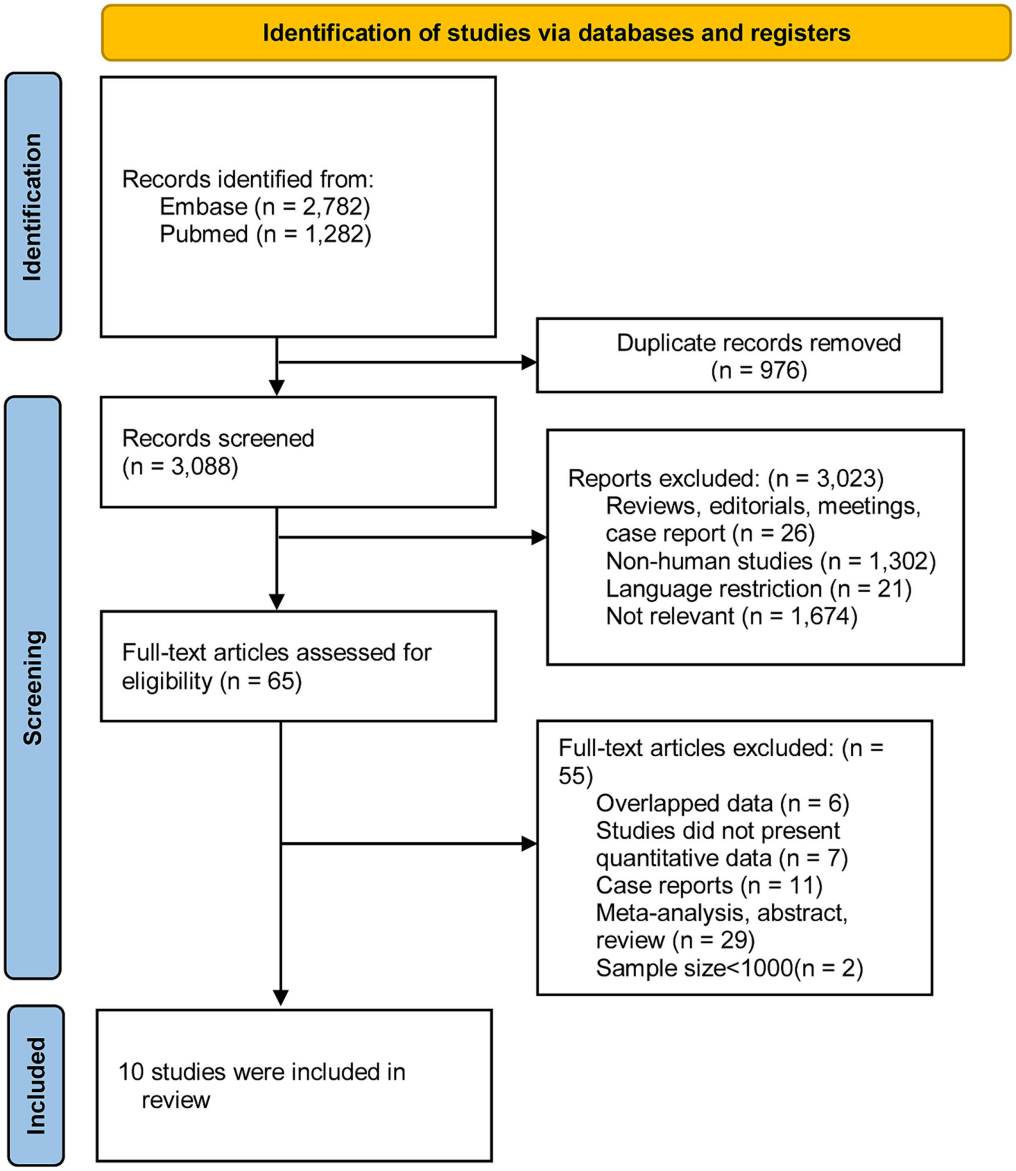
Flow-chart of the studies considered and finally selected for review.

Through keyword searches, a total of 3,088 citations were retrieved from two databases, following the removal of duplicates. After reviewing titles and abstracts, 733 articles were deemed irrelevant and excluded. A detailed full-text review was conducted for 65 articles. Ultimately, 10 studies met the criteria and were included in our analysis. Figure 1 illustrates the excluded studies along with the reasons for their exclusion after assessment of the full text.

### Characteristics of the included studies

The principal characteristics of the 10 studies included in this review are presented in Table 2. These studies were published between 2016 and 2023. The sample sizes varied, ranging from 4,898 to 3,607,599, totaling 12,717,747 subjects. Geographically, three cohort studies were conducted in Korea and the United States, three case-control studies in Europe, China, and the United States, and four cross-sectional studies in the United States, Israel, and Sweden. Participant age was > 6 years in all studies: four studies focused on children and adolescents, one included both children and adults, while the remaining five involved only adults. For diagnosing AD, two studies relied on questionnaires or medical record reviews, and four used the International Classification of Diseases (ICD) criteria. In a study by Fuxench et al., AD diagnosis was based on at least one of five specific AD-diagnostic codes and two AD-treatment codes. Another study identified AD cases through prescription records of topical agents or at least three diagnoses using specific ICD-10 codes. The criteria for headache disorder diagnosis varied considerably: four studies used questionnaires or medical records, and five used ICD criteria. One study diagnosed headache disorders through diagnostic codes.

**Table 2:** Meta-analysis for studies included in the analysis.

Subgroup analysis	Number of studies	Number of estimates	Pooled OR (95% CI), I^2^ statistics (%), *P*-value for the heterogeneity Q test	Model used
Headache disorder	10	16	1.46 (1.36–1.56); I^2^ = 98.1%, *P* < 0.001	Random effects
Study design				
Cross-section	4	8	1.67 (1.5–1.86); I^2^ = 89.5%, *P* < 0.001	Random effects
Case-control	4	4	1.5 (1.07–2.08); I^2^ = 97.1%, *P* < 0.001	Random effects
Cohort	3	4	1.2 (1.13–1.27); I^2^ = 97.6%, *P* < 0.001	Random effects
Study location				
Asia	4	5	1.39 (1.27–1.53); I^2^ = 92.2%, *P* < 0.001	Random effects
Europe	3	7	1.4 (1.28–1.52); I^2^ = 97.1%, *P* < 0.001	Random effects
North Amerca	4	4	1.53 (1.18–1.99); I^2^ = 99%, *P* < 0.001	Random effects
Age range				
Children and adolescents	6	7	1.46 (1.2–1.78); I^2^ = 98.2%, *P* < 0.001	Random effects
Adults	6	9	1.47 (1.34–1.61); I^2^ = 98%, *P* < 0.001	Random effects
Study quality				
High quality	6	7	1.33 (1.22–1.45); I^2^ = 98.9%, *P* < 0.001	Random effects
Low quality	4	8	1.61 (1.47–1.75); I^2^ = 69%, *P* = 0.002	Random effects
Headache measurement				
Questionnaires or medical review	4	8	1.61 (1.47–1.75); I^2^ = 69%, *P* = 0.002	Random effects
Diagnostic code	6	7	1.33 (1.22–1.45); I^2^ = 98.9%, *P* < 0.001	Random effects
Adjusting for at least 10 variables	5	5	1.34 (1.22–1.48); I^2^ = 99.1%, *P* < 0.001	Random effects
Severity of AD				
Mild	4	6	1.26 (1.18–1.34); I^2^ = 95.4%, *P* < 0.001	Random effects
Moderate or severe	4	9	1.23 (1.15–1.32); I^2^ = 94.7%, *P* < 0.001	Random effects
Migraine	6	7	1.32 (1.18–1.47); I^2^ = 98.9%, *P* < 0.001	Random effects
Study design				
Cross-section	1	1	1.89 (1.8–1.99)	Random effects
Case-control	2	2	1.41 (0.94–2.12); I^2^ = 98.3%, *P* < 0.001	Random effects
Cohort	3	4	1.17 (1.08–1.27); I^2^ = 97.6%, *P* < 0.001	Random effects
Study location				
Asia	3	3	1.38 (1.23–1.55); I^2^ = 95.5%, *P* < 0.001	Random effects
Europe	1	2	1.09 (1.01–1.19); I^2^ = 96%, *P* < 0.001	Random effects
North Amerca	2	2	1.47 (0.91–2.4); I^2^ = 99.6%, *P* < 0.001	Random effects
Age range				
Children and adolescents	2	2	1.37 (1.14–1.65); I^2^ = 97.9%, *P* < 0.001	Random effects
Adults	4	4	1.47 (1.34–1.61); I^2^ = 98%, *P* < 0.001	Random effects
Sex				
Male	3	3	1.46 (1.18–1.81); I^2^ = 92.7%, *P* < 0.001	Random effects
Female	3	3	1.52 (1.35–1.72); I^2^ = 76.5%, *P* < 0.001	Random effects
Adjusting for at least 10 variables	4	4	1.29 (1.12–1.47); I^2^ = 99.1%, *P* < 0.001	Random effects

Regarding methodological quality, assessed using Supplementary Tables S1, S2, six studies were classified as high quality, and four studies were of low quality. The average quality score for the 10 included studies was 6.9.

### Meta-analysis

#### Headache disorder

**Figure 2:**
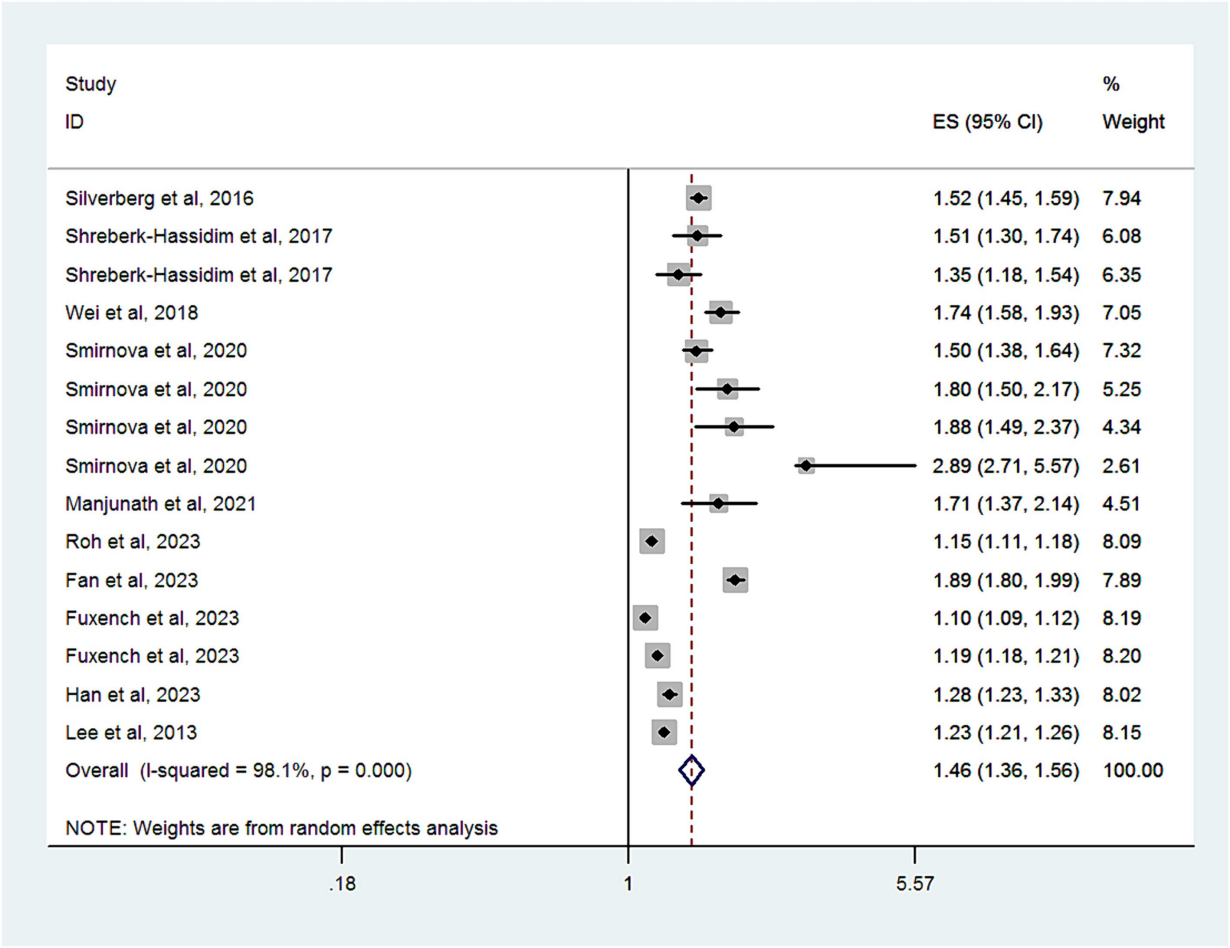
Forest plot of the overall risk of headache disorders in relation to AD.

Ten studies with a total of 12,717,747 subjects, provided data for analyzing the risk of headache disorder in patients with AD. Figure 2 demonstrates significant heterogeneity among these studies (I^2^ = 98.1%). The combined OR was 1.46 (95% CI, 1.36–1.56), suggesting a markedly increased risk of headache disorder in patients with AD. As shown in Supplementary Figure S1, there was no evidence of publication bias (Egger’s test, *p* = 0.06).

Subgroup analyses are summarized in Table 2. When categorized by study design, significant associations were found in cross-sectional studies (OR, 1.67; 95% CI = 1.5–1.86; *P* < 0.001; I^2^ = 89.5%), cohort studies (OR, 1.2; 95% CI = 1.13–1.27; *P* < 0.001; I^2^ = 97.6%), and case-control studies (OR, 1.5; 95% CI = 1.07–2.08; *P* < 0.001; I^2^ = 97.1%).

When grouped by study location, significant associations were observed in studies from Asia (OR, 1.39; 95% CI = 1.27–1.53; *P* < 0.001; I^2^ = 92.2%), Europe (OR, 1.4; 95% CI = 1.28–1.52; *P* < 0.001; I^2^ = 97.1%), and North America (OR, 1.53; 95% CI = 1.18–1.99; *P* = 0.001; I^2^ = 99%).

In a subgroup analysis of study quality, both high-quality studies (OR, 1.61; 95% CI = 1.47–1.75; *P* < 0.001; I^2^ = 69%) and low-quality studies (OR, 1.33; 95% CI = 1.22–1.45; *P* < 0.001; I^2^ = 98.9%) showed significant associations.

Age-based subgroup analysis indicated significant associations in both children and adolescents (OR, 1.46; 95% CI = 1.2–1.78; *P* = 0.001; I^2^ = 98.2%) and adults (OR, 1.47; 95% CI = 1.34–1.61; *P* < 0.001; I^2^ = 98%).

In a subgroup analysis by headache measurement, a significant association was observed in those studies using questionnaires or medical review (OR, 1.61; 95% CI = 1.47–1.75; *P* < 0.001; I^2^ = 69%) and using diagnostic code (OR, 1.33; 95% CI = 1.22–1.45; *P* < 0.001; I^2^ = 98.9%).

Four studies that assessed the impact of AD severity on headache disorder risk found a higher risk among patients with mild (OR, 1.26; 95% CI = 1.18–1.34; *P* < 0.001; I^2^ = 95.4%) or moderate to severe AD (OR, 1.23; 95% CI = 1.15–1.32; *P* < 0.001; I^2^ = 94.7%).

When we limited our analysis to studies adjusting for at least 10 variables, a significant positive association between headache disorder and AD risk was observed (OR, 1.34; 95% CI = 1.22–1.45; *P* < 0.001; I^2^ = 99.1%).

#### Migraine

**Figure 3:**
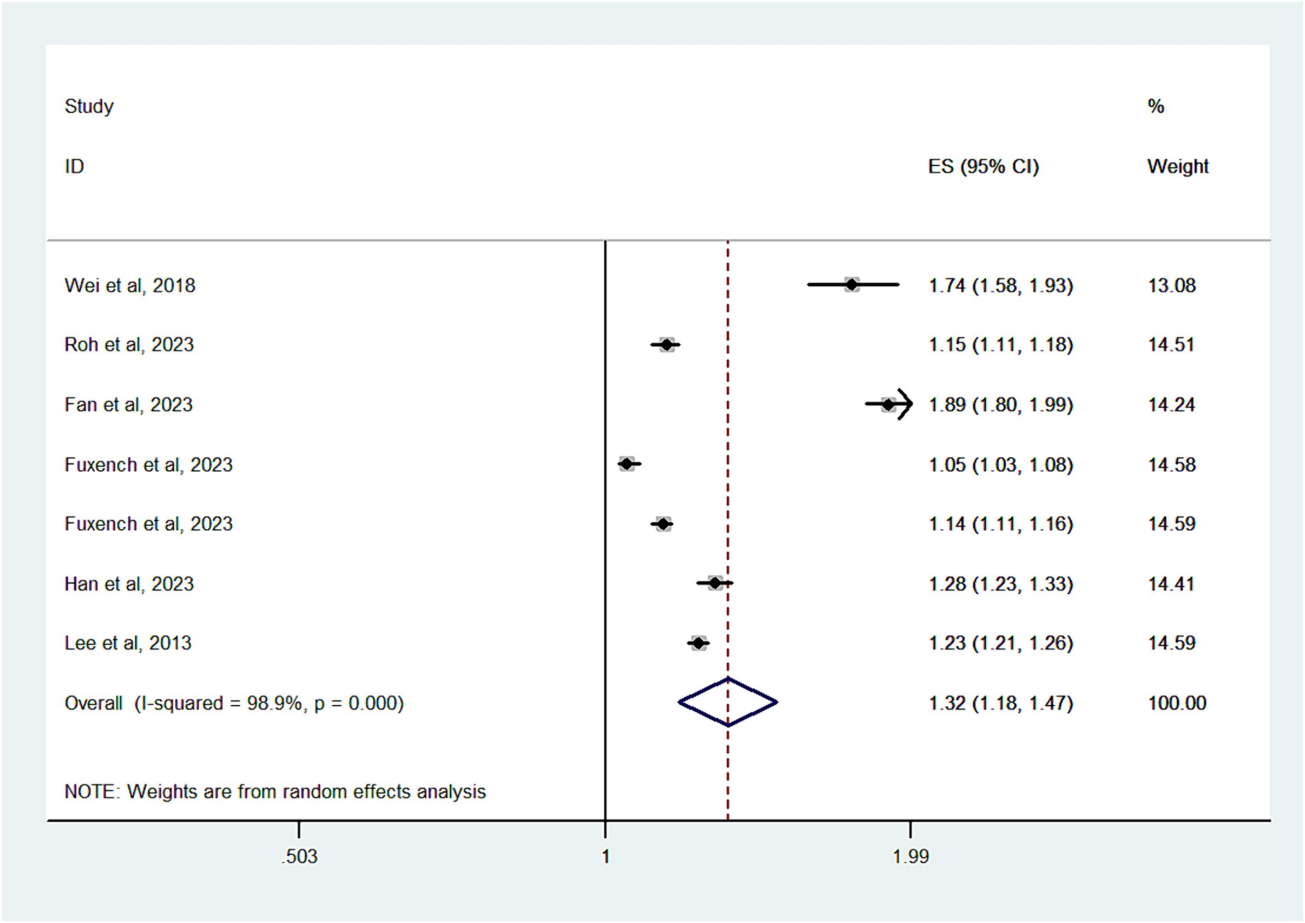
Forest plot of the overall risk of Migraine in relation to AD.

Six studies with a total of 11,090,412 subjects, contributed data for the analysis of migraine risk in patients with AD. Substantial heterogeneity was noted (I^2^ = 98.9%) (Figure 3). The overall OR was 1.32 (95% CI, 1.18–1.47), indicating a significantly increased risk of migraine in patients with AD. As shown in Supplementary Figure S2, there was no evidence of publication bias (Egger’s test, *p* = 0.6).

In a subgroup analysis by study design, significant associations were found in cross-sectional studies (OR, 1.89; 95% CI = 1.8–1.99) and cohort studies (OR, 1.17; 95% CI = 1.08–1.27; *P* < 0.001; I^2^ = 97.6%). However, case-control studies did not show an increased risk of migraine (OR, 1.41; 95% CI = 0.94–2.12; *P* = 0.097; I^2^ = 98.3%).

Grouping by study location revealed significant associations in studies from Asia (OR, 1.38; 95% CI = 1.23–1.55; *P* < 0.001; I^2^ = 95.5%) and Europe (OR, 1.09; 95% CI = 1.01–1.19; *P* < 0.001; I^2^ = 96%). Studies from North America, however, did not show an increased risk (OR, 1.47; 95% CI = 0.91–2.4; *P* = 0.12; I^2^ = 99.6%).

In an age-based subgroup analysis, a significant association was observed in adults (OR, 1.31; 95% CI = 1.16–1.48; *P* < 0.001; I^2^ = 98.9%), but not in children and adolescents (OR, 1.35; 95% CI = 0.82–2.21; *P* = 0.237; I^2^ = 98.9%).

Regarding sex, significant associations were observed in both males (OR, 1.52; 95% CI = 1.35–1.72; *P* = 0.001; I^2^ = 92.7%) and females (OR, 1.46; 95% CI = 1.18–1.81; *P* < 0.001; I^2^ = 92.7%).

When we limited our analysis to studies adjusting for at least 10 variables, a significant positive association between migraine and AD risk was observed (OR, 1.29; 95% CI = 1.12–1.47; *P* < 0.001; I^2^ = 99.2%).

## Discussion

The present study revealed that AD is associated with a moderately increased risk of headache disorder or migraine. This finding was consistent across various subgroup meta-analyses.

To our knowledge, this is the first meta-analysis to assess the association between AD and headache disorder. The fairly frequent coexistence of migraine and allergic diseases was well described in previous epidemiologic studies (30, 31). While recent meta-analyses (11–13) have indicated a bidirectional association between asthma and headache disorder, our study extends this understanding. The concept of the atopic march describes the progression of atopic disorders from AD in infancy to allergic rhinitis and asthma in childhood (32). Therefore, it is plausible that AD might also elevate the risk of headache disorder. The mechanisms linking AD and migraine could involve inflammatory responses and psychological stress triggers. Prior research has identified elevated levels of histamine, leukotrienes, and prostaglandins in migraine patients (33–35), which are crucial immune modulators in atopic disorders. Additionally, one study reported fluctuating levels of IL-10, IL-4, and IL-5 during headache attacks, suggesting a role for TH2 inflammation in both AD and headache disorders (36, 37). Moreover, a significant portion of AD patients suffer from psychiatric disorders such as depression, anxiety, and insomnia (38), which are known to have a bidirectional relationship with headache disorders. A recent extensive genome-wide study also indicated a genetic correlation between headache disorders and psychiatric conditions (39), hinting at potential common genetic factors or shared pathways.

However, the general conclusion of our study is not definitive due to considerable heterogeneity. This clinical heterogeneity is likely to have contributed to statistical heterogeneity, potentially arising from differences in study design, definitions of headache disorders or AD, age of subjects, and severity of AD. We conducted subgroup analyses based on these factors to investigate potential sources of heterogeneity. Notably, the pooled estimate from cross-sectional studies was the highest, possibly owing to inherent limitations of this study design. Cross-sectional studies allow categorization of subjects by previous exposure at a specific time point, but they can also introduce recall bias. This bias may occur because the presence of a disease or its assessment could influence participants’ responses, potentially leading to an overestimation of associations.

Furthermore, the varied definitions of headache disorder or AD may contribute to the heterogeneity observed in our findings. Several studies included in our analysis relied on questionnaires to confirm cases of headache disorder or AD, potentially impacting the accuracy and completeness of diagnoses. Consequently, misclassification of headache disorder or AD might weaken the robustness of our results. However, the results from subgroup analyses based on diagnostic criteria were still consistent with the overall analysis. However, the pooled OR of studies using diagnostic code was lower, indicating that the unreliability of the diagnoses may overestimate this association. Prior study has shown a positive association between inflammatory cytokines and the severity of AD (40), leading to the hypothesis that the risk of headache disorders could vary with the severity of AD. Interestingly, our study did not find a severity-dependent association between AD and headache disorder, but this finding is limited by the sample size and necessitates further investigation.

The strength of our study lies in its large sample size and extensive search methodology, which enabled a detailed examination of the association. Additionally, we conducted comprehensive subgroup analyses based on various factors such as study region and design, type of headache disorder, measurement methods for headache disorder or AD, study quality, severity of AD, and gender. However, there are several significant limitations to consider. The primary limitation is the potential influence of unknown confounders. As we known, the pathogenesis of the headache disorder is complex and related to many factors. Therefore, only adjusted risk estimates were included in our meta-analysis. Furthermore, we conducted analyses limited to studies adjusting at least 10 variables and the pooled ORs were reduced but still significant. More well-designed studies that consider additional important covariates are needed. Second, the limited number of eligible high-quality studies may affect the precision of our findings. Third, there is a lack of data regarding the impact of medications for AD on the risk of headache disorder. A previous study (41) suggested that anti-asthmatic or anti-allergic treatments might reduce migraine risk in children and adolescents. Future research should include consideration of anti-allergic drug use to clarify further the link between AD and headache disorders.

In conclusion, our results indicate that AD may be a potential risk factor for headache disorder. Clinicians should be mindful of the increased incidence of headache disorder in patients with AD. To examine these findings further and gain a more comprehensive understanding, future prospective cohort studies with more comprehensive data collection and consideration of potential confounders are necessary to provide additional evidence on the detailed association.

## Data availability statement

The original contributions presented in the study are included in the article/Supplementary material, further inquiries can be directed to the corresponding author.

## Author contributions

WY: Writing – review & editing, Data curation, Methodology, Software, Visualization. HD: Conceptualization, Formal analysis, Investigation, Methodology, Project administration, Software, Validation, Writing – review & editing. X-fX: Writing – review & editing, Formal analysis, Project administration, Resources, Supervision. H-yJ: Supervision, Validation, Writing – original draft, Writing – review & editing. J-yD: Resources, Software, Supervision, Validation, Writing – original draft, Writing – review & editing.
