# Supplementary material for: Association of atopic dermatitis and headache disorder: a systematic review and meta-analyses
Source: Front Neurol. 2024 Mar 21;15:1383832. doi: 10.3389/fneur.2024.1383832 (PMC10991782; doi:10.3389/fneur.2024.1383832)

| **Table S1** NOS for Assessment of Quality of Included Studies: Case-Control or corss-sectional Studies | | | | | | | | |
| --- | --- | --- | --- | --- | --- | --- | --- | --- |
| Study | Selection | | | | Comparability | Exposure | | |
|  | Is the case definition adequate | Representativeness of cases | Selection of controls | Definition of controls | Study controls for at least 3 additional factors | Ascertainment of exposure | Same method of ascertainment of exposure | Nonresponse rate |
| Silverberg et al, 2016 | ★ | **—** | ★ | ★ | ★★ | **—** | ★ | — |
| Shreberk-Hassidim et al, 2017 | ★ | — | ★ | ★ | ★ | ★ | ★ | — |
| Wei et al, 2018 | ★ | **—** | ★ | ★ | ★ | **★** | ★ | **★** |
| Smirnova et al, 2020 | ★ | — | ★ | ★ | ★ | ★ | ★ | — |
| Manjunath et al, 2021 | ★ | — | — | ★ | ★ | ★ | ★ | — |
| Roh et al, 2023 | ★ | **—** | ★ | ★ | ★ | **★** | ★ | **★** |
| Fan et al, 2023 | ★ | **—** | ★ | ★ | ★★ | **★** | ★ | **★** |

| **Table S2**  NOS for Assessment of Quality of Included Studies: Cohort Studies | | | | | | | | |
| --- | --- | --- | --- | --- | --- | --- | --- | --- |
| Study | Selection | | | | Comparability | | Outcomes | |
|  | Representativeness of exposed cohort? | Selection of the nonexposed cohort? | Ascertainment of exposure? | Demonstration that outcome of interest was not represent at the start of the study | Comparability of Cohort* | Assessment of outcome | Was follow-up long enough for outcomes to occur | Adequacy of follow up of cohorts |
| Fuxench et al, 2023 | ★ | ★ | ★ | ★ | ★★ | ★ | ★ | — |
| Han et al, 2023 | ★ | ★ | ★ | ★ | ★★ | ★ | ★ | — |
| Lee et al, 2013 | ★ | ★ | ★ | ★ | ★★ | ★ | ★ | — |
| Note: A star denotes a score of 1; * A maximum of 2 stars can be allotted in this category | | | | | | | | |

**Figure S1** The Egger’s test for identifying publication bias in a meta-analysis of observational studies evaluating the association between AD and headache disorders.


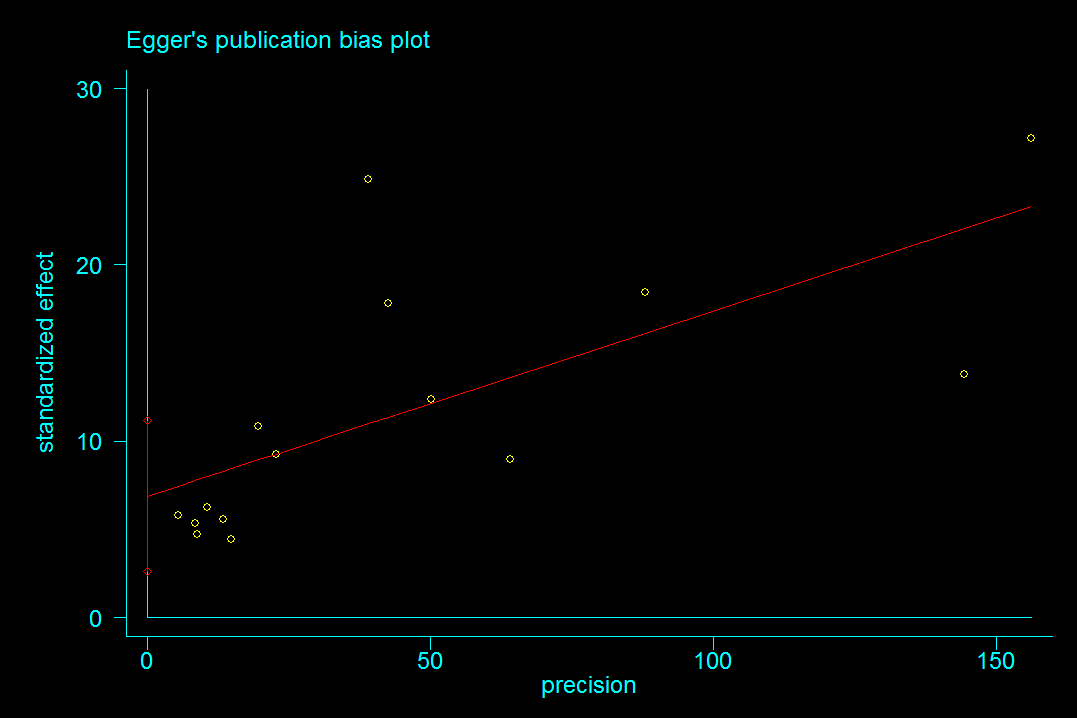


**Figure S2** The Egger’s test for identifying publication bias in a meta-analysis of observational studies evaluating the association between AD and migraine.


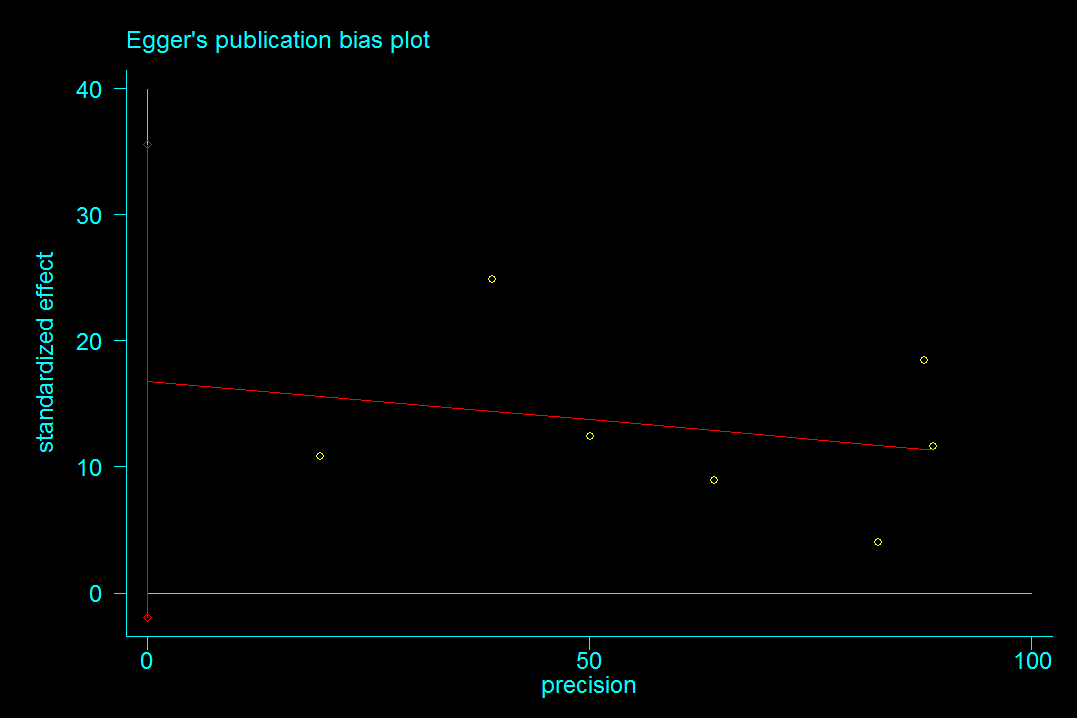

Supplement: Supplementary file 1 [file Data_Sheet_1.docx]
